# Pulpotomy for an Immature Permanent Tooth With Symptomatic Irreversible Pulpitis Using Cold Ceramic

**DOI:** 10.1155/crid/4509890

**Published:** 2026-07-20

**Authors:** Narges Farhad Mollashahi, Alireza Ansari, Amir Hossein Khazaei

**Affiliations:** ^1^ Department of Endodontics, School of Dentistry, Zahedan University of Medical Sciences, Zahedan, Iran, zaums.ac.ir; ^2^ School of Dentistry, Zahedan University of Medical Sciences, Zahedan, Iran, zaums.ac.ir

**Keywords:** biomaterial, case report, immature permanent tooth, irreversible pulpitis, pulpotomy

## Abstract

**Background:**

With recent advances in biomaterials, there has been growing interest in using pulpotomy to treat irreversible pulpitis instead of root canal therapy. In this report, we describe a case of pulpotomy with 9‐month follow‐up in an immature permanent tooth exhibiting signs and symptoms of symptomatic irreversible pulpitis.

**Case Description:**

A 9‐year‐old female presented with a chief complaint of a severe toothache in the lower left molar. Radiologic examination revealed a deep carious lesion and Nolla Stage 9. Based on the findings, a symptomatic irreversible pulpitis was diagnosed. Pulpotomy was preferred over root canal therapy in order to preserve the pulp and allow the roots to continue developing. Cold ceramic was used as a biomaterial barrier during pulpotomy. Evidence of dentin bridge formation, root development, and normal responses to cold and electric pulp tests were observed during the follow‐up sessions at 3, 6, and 9 months.

**Conclusion:**

Pulpotomy can be a good treatment option for irreversible pulpitis in immature permanent teeth. Irreversible pulpitis in these teeth does not necessarily mean that the entire pulp has been affected. By removing only the affected part of the pulp with pulpotomy, the healthy pulp could still be preserved.

## 1. Introduction and Importance

When tooth decay is left untreated, it can lead to a serious condition called irreversible pulpitis.

This happens when the dental pulp becomes inflamed due to caries or injury. Untreated irreversible pulpitis in young permanent teeth can cause problems like thinning of the root canal walls, an imbalance between the crown and root size, and arrested tooth development [1]. However, there are no clear guidelines for treating irreversible pulpitis in immature permanent teeth [2, 3].

The American Association of Endodontists (AAE) suggests a total pulpectomy and root canal filling for irreversible pulpitis [4]. Another method is apexification, which is used for immature teeth [5]. However, both these methods involve removing the entire radicular pulp, which could lead to a higher risk of secondary root fracture. For reversible pulpitis in permanent teeth, like in cases of asymptomatic carious or traumatic pulp exposure, a procedure called pulpotomy can be a good alternative [6]. Young permanent teeth have a larger apical foramen and pulp tissue at the apex that moves with the periapical tissues. These teeth also have good vascularization, making their pulp more resistant to inflammation [7]. This gives them a natural ability for vital pulp therapy, which is a key reason why we might try to preserve the pulp instead of removing it completely [7].

In recent years, there has been growing interest in using pulpotomy to treat irreversible pulpitis.

New materials and better understanding of how pulp works have improved the success of pulpotomy in mature teeth, even for conditions like irreversible pulpitis. However, data regarding the outcomes of pulpotomy in immature permanent teeth are still little [8]. In this report, we described a case of pulpotomy with 9‐month follow‐up in an immature permanent tooth exhibiting signs and symptoms of symptomatic irreversible pulpitis. The clinical significance of this report is its valuable images showing dentin bridge formation and root development after 9‐month follow‐up. In addition, we used cold ceramic (CC) as a biomaterial barrier for this case. To the best of our knowledge, no other study has reported its usage in a tooth with a similar condition and diagnosis. This material is a newly developed bioceramic with comparable clinical properties with other calcium silicate–based cements, such as mineral trioxide aggregate (MTA) and Biodentine, and has a lower cost [9]. This case report has been reported in line with the CARE checklist.

## 2. Presentation of Case

### 2.1. Patient Information, Clinical Findings, and Diagnostic Assessment

A 9‐year‐old female was referred from a pedodontist to the Department of Endodontics, Zahedan Faculty of Dentistry, for root canal treatment of an immature permanent first molar. The chief complaint of the patient was severe toothache of the lower left molar. This patient had undergone dental treatments before and therefore was acquainted with the dental environment. A thorough assessment of past medical, family, and social history did not reveal any significant issues. The patient had no systemic disease and was not consuming any medications. No allergies were reported in this patient. Dental history revealed no trauma to the tooth.

Intraoral examination showed positive (++) cold and electric pulp tests, different sensation on percussion and palpation, and normal mobility of the lower left molar. No tooth discoloration was observed. A severe spontaneous pain was also reported by the patient with a sustained pain after cold and heat stimulation. Radiologic examination showed a deep carious lesion and Nolla Stage 9 (root length almost complete with parallel ends, and open apex with radiolucency and wide periodontal ligament space) [10] (Figure 1A). Based on these findings, a symptomatic irreversible pulpitis was diagnosed in this tooth. The AAE diagnostic criteria for pulpal disease is presented in Table 1 [11].

**Figure 1:**
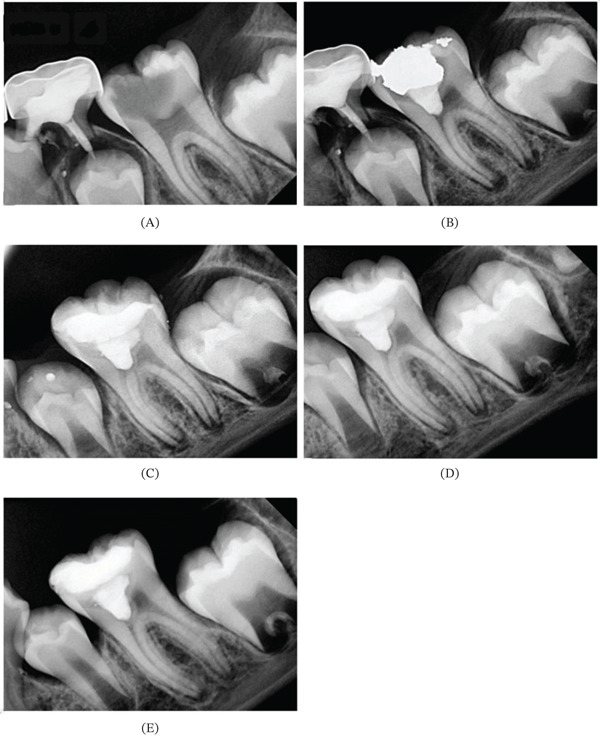
Radiological examination of the lower left molar (Tooth #36) in a 9‐year‐old female. (A) Deep carious lesion leading to symptomatic irreversible pulpitis. (B) Amalgam filling as a temporary restoration after pulpotomy of the tooth using cold ceramic as a biomaterial barrier. (C) Early signs of dentin bridge formation after 3 months. (D) Dentin bridge formation after 6 months of treatment. (E) After 9 months, the development of roots was evident.

**Table 1:** The American Association of Endodontists diagnostic criteria for pulpal disease.

Diagnosis category	Reversible pulpitis	Symptomatic irreversible pulpitis	Asymptomatic irreversible pulpitis	Necrotic pulp
Pain	Pain upon stimulation but reverses quickly after removal of stimulus.	Lingering pain, spontaneous pain, referred pain, pain on postural changes, pain despite using analgesic.	No pain	Often no pain unless the inflammation has spread to the apical tissues.
Diagnostic tests	Cold sensitivity goes away quickly (within 1–2 s).	Cold test: pain persists for more than 10–30 s after removing the cold source.	Diagnosis is made via clinical findings where the pulp is exposed (e.g., due to caries or trauma) but the patient does not report symptoms.	No response to cold or electric pulp tests.
		Heat test: intense pain that lingers.		
		Percussion/palpation: usually negative unless the inflammation has spread to the apical tissues.		
		Radiographs: often show deep caries or extensive restorations/fractures approaching the pulp chamber.		

### 2.2. Therapeutic Intervention

Due to the significant impact of root development on the prognosis of the tooth, pulpotomy was carried out in this case by an endodontist with more than 2 years of experience.

The patient was given local anesthesia using an inferior alveolar nerve block and local infiltration with lidocaine 2% + 1:80,000 epinephrine hydrochloride (Darou Pakhsh, Tehran, Iran). A rubber dam was used to protect the area. A fast emery drill was used to remove the enamel and a slow‐speed tungsten steel ball drill to remove the dentin and expose the pulp. The dentist first checked the pulp′s condition and any bleeding. The texture (liquefied), color (dark red), and excessive bleeding upon exposure indicated an unhealthy pulp. Then, a sterile high‐speed diamond ball drill was used to partially remove the coronal pulp. The amount of pulp removed depended on how much bleeding occurred. If the bleeding could not be controlled after removing part of the pulp, more pulp would be removed. Next, 5.25% sodium hypochlorite was used to rinse the area, and a sterile saline cotton ball was placed in the pulp for 3–5 min to help stop the bleeding. Once the cotton was removed, the area without active bleeding was considered to have good control of bleeding. Bright red color and homogeneous texture were other indicators of healthy pulp. Then, CC (SJM, Iran) of 2–3 mm was applied.

Due to financial challenges, the patient could not afford the widely studied biomaterials such as MTA and Biodentine. Therefore, we decided to use CC to address this challenge. The ideal qualities of CC including shorter setting time than MTA, a better seal than glass ionomer, ideal biocompatibility and nontoxicity, and favorable radiopacity led us to select this material [12, 13].

Light‐cure glass ionomer (Fuji II, GC, Japan) was used as a base, and a temporary amalgam restoration (Cinalux: Owzan Company, Iran) was used to restore the tooth (Figure 1B). At the end of treatment, oral hygiene was reinforced, and the patient was asked to report any discomfort until the next follow‐up.

### 2.3. Follow‐Up and Outcomes

Three months later, the patient was asked for follow‐up and final restoration with composite resin (Tetric N‐Ceram, Ivoclar Vivadent, Liechtenstein). The patient had no signs and symptoms on the follow‐up session. Cold and electric pulp tests showed normal findings. The radiologic examination of 3‐month follow‐up is shown in Figure 1C. Early signs of dentin bridge formation was evident. Again, oral hygiene was reinforced, and the patient was asked to come 3 months later. The radiologic examination of 6‐month follow‐up is shown in Figure 1D. Dentin bridge formation was evident, and the pulp tests were normal. After 9 months, the development of roots was evident as shown in Figure 1E. Therefore, dentin bridge formation, root development, and normal pulp tests were evident in this case 9 months after the therapeutic intervention. Patient reported no complications except a mild pain on the first day of treatment.

## 3. Clinical Discussion

The pain that persists indicates the possibility of irreversible pulpitis; however, this does not necessarily mean that the entire pulp has been irreversibly damaged [14]. Research in the last few decades has focused on the treatment of irreversible pulpitis by less invasive methods, aiming to preserve the natural function of dental pulp [14]. Despite initial claims that clinical signs and symptoms may not accurately reflect the actual histopathological condition of the pulp, new research has now confirmed the apparent correlation between clinical signs and symptoms and the histopathological condition of the pulp [14]. The AAE diagnostic classification is based on signs and symptoms. Although histological examination is considered the gold standard diagnostic test for pulpal disease, it is not feasible if pulp preservation is indicated. Wolters et al. [15] described a new classification focusing on the possible pulp preservation therapies for each category. Accordingly, pulpitis is categorized into initial, mild, moderate, and severe pulpitis. The suggested treatments for each category are as follows: indirect pulp treatment (IPT) for initial and mild pulpitis; partial or full pulpotomy for moderate pulpitis; and full pulpotomy or pulpectomy (depending on bleeding control) for severe pulpitis [15]. Although Karrar et al. [16] have shown the clinical validity of this classification, further investigation is required to assess the clinical outcomes of each suggested treatment for each category.

Different biomaterials have been used in the pulpotomy of immature permanent teeth with irreversible pulpitis. CC is a calcium hydroxide–based material with main components of calcium oxide, silicon oxide, barium oxide, and sulfur trioxide [9]. To the best of our knowledge, no previous similar study has reported the use of CC in the pulpotomy of an immature permanent tooth with irreversible pulpitis. Several studies have evaluated its application in other types of treatments. Rasteh et al. [12] showed that CC has favorable clinical outcomes in pulpotomy therapy of primary teeth. This material showed promising results in the treatment of root perforations according to the studies by Chamani et al. [17] and Modaresi et al. [18].

The most common used material for pulpotomy of immature permanent teeth with irreversible pulpitis is MTA [19]. MTA exhibits superior biocompatibility and sealing properties compared with calcium hydroxide, which can promote cell growth in the dental pulp and result in a thicker, more complete dentin bridge with fewer defects. Consequently, employing MTA as a barrier material can enhance the efficacy of this procedure [19]. An earlier investigation revealed that the continuity and shape characteristics of dentin bridge structures formed using Vitapex, a material composed primarily of calcium hydroxide, iodoform, and silicone oil, exhibited inferior results compared with those induced by MTA [20]. However, MTA has certain limitations. Among them, tooth discoloration remains an important factor affecting patient quality of life. Biodentine, a dentin substitute derived from MTA, can achieve high clinical and radiographic success rates with a lower rate of tooth discoloration compared with MTA when employed as a barrier for vital pulp therapy in young permanent teeth [21].

Sodium hypochlorite efficiently removes debris and bacterial contamination while also aiding in hemostasis [22]. In addition, sodium hypochlorite does not pose any toxicity to pulp tissue and does not impair the regeneration of dental pulp; consequently, it has been widely used in pulpotomy [22]. However, the amount of time needed to achieve hemostasis through this procedure is still controversial [2, 19, 23]. Therefore, in order to make sure that all of the inflammatory tissue was removed, it was recommended to examine the pulp directly under a dental operating microscope [24].

The dentin bridge formation on the treated pulp section indicates viability and pulp healing capacity. Dentin bridge formation has been proposed as a key sign of successful treatment [25]. However, a number of studies have shown that the treatment success of immature permanent teeth with carious pulp exposure was also achieved in several teeth without dentin bridge formation [26, 27].

In our case report, the patient had irreversible pulpitis induced by a carious lesion. The cause of irreversible pulpitis appears to be a factor in the success of pulpotomy. In the study by Zhang et al. [27], the treatment success rate of pulpotomy for traumatic crown fracture was lower than for dental caries and dens evaginatus. The disruption of the pulp blood supply in the apical region and potential damage to periodontal tissue may be the cause of this finding [28]. However, immediate pulpotomy after trauma can achieve a higher success rate compared with delayed pulpotomy [29].

Zhang et al. [27] reported that the success rate of pulpotomy in Nolla Stage 9 teeth was similar to that in Nolla Stages 7 and 8. Therefore, it appears that the impact on the treatment outcomes did not depend upon the degree of tooth‐root maturation, and this finding aligns with earlier research [26, 30].

## 4. Conclusion

Pulpotomy can be a good treatment option for irreversible pulpitis in immature permanent teeth.

Irreversible pulpitis in these teeth does not necessarily mean that the entire pulp has been affected. By removing only the affected part of the pulp with pulpotomy, the healthy pulp could still be preserved. In this case report, we utilized CC as a barrier and recorded promising outcomes after 9 months in the pulpotomy of a carious immature mandibular molar (Nolla Stage 9) with signs and symptoms of symptomatic irreversible pulpitis. However, further investigation is required to validate these findings and address the limitations with a larger sample size and longer follow‐up period.

## Author Contributions

Narges Farhad Mollashahi: conceptualization, methodology, supervision. Alireza Ansari: project administration, validation, investigation. Amir Hossein Khazaei: project administration, writing—original draft, writing—review and editing, validation, investigation, visualization.

## Funding

No funding was received for this manuscript.

## Disclosure

All authors have read and approved the final version of the manuscript. The corresponding author had full access to all of the data in this study and takes complete responsibility for the integrity of the data and the accuracy of the data analysis.

## Ethics Statement

This case report was performed in line with the principles of the Declaration of Helsinki. Institutional Review Board (IRB) approval was granted by the Ethics Committee of Zahedan University of Medical Sciences (IR.ZAUMS.REC.1404.349).

## Consent

Informed consent was obtained from the patient′s legal guardians.

## Conflicts of Interest

The authors declare no conflicts of interest.

## Patient Perspective

The patient and her family reported mild pain on the first day. However, after taking a capsule of Megafen (acetaminophen 325 mg/caffeine 30 mg/ibuprofen 200 mg) the pain was relieved. It was recommended that the patient take this medicine every 8 h on the first day. No pain was reported on the second day. The patient did not report any other complications.

## Supporting information


**Supporting Information** Additional supporting information can be found online in the Supporting Information section. The filled CARE checklist.

## Data Availability

Data sharing is not applicable to this article as no datasets were generated or analyzed during the current study.

## References

[1] Dean J. , Avery D. , and Mcdonald R. , McDonald′s and Avery′s Dentistry for the Child and Adolescent, 2021, 9th edition, Elsevier.

[2] Bogen G. and Chandler N. P. , Pulp Preservation in Immature Permanent Teeth, Endodontic Topics. (2010) 23, no. 1, 131–152, 10.1111/j.1601-1546.2012.00286.x.

[3] American Academy of Pediatric Dentistry , Pulp Therapy for Primary and Immature Permanent Teeth, 2020, The Reference Manual of Pediatric Dentistry.

[4] Ather A. , Patel B. , Gelfond J. A. L. , and Ruparel N. B. , Outcome of Pulpotomy in Permanent Teeth With Irreversible Pulpitis: A Systematic Review and Meta-Analysis, Scientific Reports. (2022) 12, no. 1, 19664, 10.1038/s41598-022-20918-w, 36385132. 36385132 PMC9669040

[5] Huang G. T. , Apexification: The Beginning of Its End, International Endodontic Journal. (2009) 42, no. 10, 855–866, 10.1111/j.1365-2591.2009.01577.x, 19549154. 19549154

[6] Uyar D. S. and Alacam A. , Evaluation of Partial Pulpotomy Treatment in Cariously Exposed Immature Permanent Molars: Randomized Controlled Trial, Nigerian Journal of Clinical Practice. (2021) 24, no. 10, 1511–1519, 10.4103/njcp.njcp_686_20, 34657018. 34657018

[7] Farges J. C. , Alliot-Licht B. , Renard E. , Ducret M. , Gaudin A. , Smith A. J. , and Cooper P. R. , Dental Pulp Defence and Repair Mechanisms in Dental Caries, Mediators of Inflammation. (2015) 2015, no. 1, 230251, 10.1155/2015/230251, 26538821. 26538821 PMC4619960

[8] Qudeimat M. A. , Alyahya A. , and Hasan A. A. , Mineral Trioxide Aggregate Pulpotomy for Permanent Molars With Clinical Signs Indicative of Irreversible Pulpitis: A Preliminary Study, International Endodontic Journal. (2017) 50, no. 2, 126–134, 10.1111/iej.12614, 26841969. 26841969

[9] Modaresi J. and Hemati H. R. , The Cold Ceramic Material, Dental Research Journal. (2018) 15, no. 2, 85–88, 10.4103/1735-3327.226522, 29576770. 29576770 PMC5858076

[10] Pliska B. , Nahvi A. , Pakdaman N. , Dadgar S. , Aryana M. , and Sobouti F. , Radiological Evaluation of the Accuracy of Demirjian, Nolla, and Willems Methods for Dental Age Estimation in 3–17-Year-Old Iranian Children, Bio Med Research International. (2024) 2024, no. 1, 8783660, 10.1155/2024/8783660. PMC11236468 38988904

[11] Glickman G. N. , AAE Consensus Conference on Diagnostic Terminology: Background and Perspectives, Journal of Endodontics. (2009) 35, no. 12, 1619–1620, 10.1016/j.joen.2009.09.029, 19932336. 19932336

[12] Rasteh B. , Basir L. , Taravati S. , and Khataminia M. , Evaluation and Comparison of Mineral Trioxide Aggregate and Cold Ceramic in Primary Tooth Pulpotomy: Clinical and Radiographic Study, Journal of Family Medicine and Primary Care. (2023) 12, no. 12, 3068–3074, 10.4103/jfmpc.jfmpc_412_23, 38361859. PMC10866251 38361859

[13] Khedmat S. , Sarraf P. , Seyedjafari E. , Sanaei-rad P. , and Noori F. , Comparative Evaluation of the Effect of Cold Ceramic and MTA-Angelus on Cell Viability, Attachment and Differentiation of Dental Pulp Stem Cells and Periodontal Ligament Fibroblasts: An In Vitro Study, BMC Oral Health. (2021) 21, no. 1, 10.1186/s12903-021-01979-1, 34876089. PMC8650362 34876089

[14] Ricucci D. , Loghin S. , and Siqueira J. F. , Correlation Between Clinical and Histologic Pulp Diagnoses, Journal of Endodontics. (2014) 40, no. 12, 1932–1939, 10.1016/j.joen.2014.08.010. 25312886

[15] Wolters W. J. , Duncan H. F. , Tomson P. L. , Karim I. E. , McKenna G. , Dorri M. , Stangvaltaite L. , and van der Sluis L. W. M. , Minimally Invasive Endodontics: A New Diagnostic System for Assessing Pulpitis and Subsequent Treatment Needs, International Endodontic Journal. (2017) 50, no. 9, 825–829, 10.1111/iej.12793, 28776717. 28776717

[16] Karrar R. N. , Craig S. G. , Duncan H. F. , Abushouk S. A. , Elfiel S. Y. , Lundy F. T. , Clarke M. , el-Karim I. A. , and Reliability Assessment Group , Clinical Validation of a Proposed Diagnostic Classification for Pulpitis, International Endodontic Journal. (2025) 58, no. 8, 1158–1171, 10.1111/iej.14254, 40423642. 40423642 PMC12254530

[17] Chamani A. , Forghani M. , and Asadi G. , Cold Ceramic for Repairing Root Perforations: A Case Report, Clinical Case Reports. (2025) 13, no. 2, e70182, 10.1002/ccr3.70182, 39949583. 39949583 PMC11821458

[18] Modaresi J. , Parashos P. , Mousavi R. , Mirzaeeian A. , and Almodaresi Z. , Treatment of Strip Perforation Using Cold Ceramic, Dental Research Journal. (2023) 20, no. 1, 10.4103/1735-3327.372648. PMC10166746 37180691

[19] Duncan H. F. , Galler K. M. , Tomson P. L. , Simon S. , El-Karim I. , Kundzina R. , Krastl G. , Dammaschke T. , Fransson H. , and Markvart M. , European Society of Endodontology Position Statement: Management of Deep Caries and the Exposed Pulp, International Endodontic Journal. (2019) 52, no. 7, 923–934, 10.1111/iej.13080, 30664240. 30664240

[20] Kwon W. , Kim I. H. , Kang C. M. , Kim B. , Shin Y. , and Song J. S. , Comparative Study of Pulpal Responses to ProRoot MTA, Vitapex, and Metapex in Canine Teeth, Journal of Dental Sciences. (2021) 16, no. 4, 1274–1280, 10.1016/j.jds.2020.12.011, 34484596. 34484596 PMC8403786

[21] Lu L. and Zheng W. , Evaluation of Vital Pulp Therapy With Biodentine in Young Permanent Teeth: A Systematic Review and Meta-Analysis, Journal of Clinical Pediatric Dentistry. (2025) 49, no. 3, 9–20, 10.22514/jocpd.2025.045.

[22] Cai C. , Chen X. , Li Y. , and Jiang Q. , Advances in the Role of Sodium Hypochlorite Irrigant in Chemical Preparation of Root Canal Treatment, BioMed Research International. (2023) 2023, no. 1, 8858283, 10.1155/2023/8858283, 36685672. 36685672 PMC9859704

[23] Ricucci D. , Siqueira J. F. , Li Y. , and Tay F. R. , Vital Pulp Therapy: Histopathology and Histobacteriology-Based Guidelines to Treat Teeth With Deep Caries and Pulp Exposure, Journal of Dentistry. (2019) 86, 41–52, 10.1016/j.jdent.2019.05.022, 31121241. 31121241

[24] Ricucci D. , Grande N. M. , Plotino G. , and Tay F. R. , Histologic Response of Human Pulp and Periapical Tissues to Tricalcium Silicate-Based Materials: A Series of Successfully Treated Cases, Journal of Dentistry. (2020) 46, no. 2, 307–317, 10.1016/j.joen.2019.10.032, 31836137. 31836137

[25] Kunert G. G. , Kunert I. R. , da Costa Filho L. C. , and de Figueiredo J. A. P. , Permanent Teeth Pulpotomy Survival Analysis: Retrospective Follow-Up, Journal of Dentistry. (2015) 43, no. 9, 1125–1131, 10.1016/j.jdent.2015.06.010, 26144188. 26144188

[26] Taha N. A. and Abdulkhader S. Z. , Full Pulpotomy With Biodentine in Symptomatic Young Permanent Teeth With Carious Exposure, Journal of Endodontics. (2018) 44, no. 6, 932–937, 10.1016/j.joen.2018.03.003, 29681479. 29681479

[27] Zhang N. , Kang Q. , and Cheng Y. , Pulpotomy for Teeth With Irreversible Pulpitis in Immature Permanent Teeth: A Retrospective Case Series Study, Scientific Reports. (2024) 14, no. 1, 10.1038/s41598-024-56975-6, 38493190. PMC10944512 38493190

[28] Wang C. , Qin M. , and Guan Y. , Analysis of Pulp Prognosis in 603 Permanent Teeth With Uncomplicated Crown Fracture With or Without Luxation, Dental Traumatology. (2014) 30, no. 5, 333–337, 10.1111/edt.12099. 24571370

[29] Matoug-Elwerfelli M. , ElSheshtawy A. S. , Duggal M. , Tong H. J. , and Nazzal H. , Vital Pulp Treatment for Traumatized Permanent Teeth: A Systematic Review, International Endodontic Journal. (2022) 55, no. 6, 613–629, 10.1111/iej.13741, 35348216. 35348216

[30] Eren B. , Onay E. O. , and Ungor M. , Assessment of Alternative Emergency Treatments for Symptomatic Irreversible Pulpitis: A Randomized Clinical Trial, International Endodontic Journal. (2018) 51, no. Supplement 3, e227–e237, 10.1111/iej.12851. 28857203

