# Supplementary material for: Pulpotomy for an Immature Permanent Tooth With Symptomatic Irreversible Pulpitis Using Cold Ceramic
Source: Case Rep Dent. 2026 Jul 20;2026:4509890. doi: 10.1155/crid/4509890 (PMC13385973; doi:10.1155/crid/4509890)
Supplement: Supplementary file 1 — Supporting Information Additional supporting information can be found online in the Supporting Information section. The filled CARE checklist. [file CRID-2026-4509890-s001.pdf]

CARE case report guidelines

CARE Checklist of information to include when writing a case report

(cc) BY-NC-ND

✔

Topic	Item	Checklist item description	Reported on Line
Title	1	The diagnosis or intervention of primary focus followed by the words "case report"	1
Key Words	2	2 to 5 key words that identify diagnoses or interventions in this case report, including "case report"	23
Abstract (no references)	3a	Introduction: What is unique about this case and what does it add to the scientific literature?	6-9
	3b	Main symptoms and/or important clinical findings	10-16
	3c	The main diagnoses, therapeutic interventions, and outcomes	10-16
	3d	Conclusion—What is the main "take-away" lesson(s) from this case?	17-20
Introduction	4	One or two paragraphs summarizing why this case is unique (**may include references**)	41-47
Patient Information	5a	De-identified patient specific information.	50-63
	5b	Primary concerns and symptoms of the patient.	50-63
	5c	Medical, family, and psycho-social history including relevant genetic information	50-63
	5d	Relevant past interventions with outcomes	50-63
Clinical Findings	6	Describe significant physical examination (PE) and important clinical findings.	50-63
Timeline	7	Historical and current information from this episode of care organized as a timeline	50-63
Diagnostic Assessment	8a	Diagnostic testing (such as PE, laboratory testing, imaging, surveys).	50-63
	8b	Diagnostic challenges (such as access to testing, financial, or cultural)	50-63
	8c	Diagnosis (including other diagnoses considered)	50-63
	8d	Prognosis (such as staging in oncology) where applicable	-
Therapeutic Intervention	9a	Types of therapeutic intervention (such as pharmacologic, surgical, preventive, self-care)	64-80
	9b	Administration of therapeutic intervention (such as dosage, strength, duration)	64-80
	9c	Changes in therapeutic intervention (with rationale)	-
Follow-up and Outcomes	10a	Clinician and patient-assessed outcomes (if available)	64-80
	10b	Important follow-up diagnostic and other test results	80-87
	10c	Intervention adherence and tolerability (How was this assessed?)	80-87
	10d	Adverse and unanticipated events	-
Discussion	11a	A scientific discussion of the strengths AND limitations associated with this case report	90-132
	11b	Discussion of the relevant medical literature **with references**.	90-132
	11c	The scientific rationale for any conclusions (including assessment of possible causes)	90-132
	11d	The primary "take-away" lessons of this case report (without references) in a one paragraph conclusion	137-142
Patient Perspective	12	The patient should share their perspective in one to two paragraphs on the treatment(s) they received	145-148
Informed Consent	13	Did the patient give informed consent? Please provide if requested	Yes ☒ No ☐
